# Targeted suppression of autoreactive CD8^+^ T-cell activation using blocking anti-CD8 antibodies

**DOI:** 10.1038/srep35332

**Published:** 2016-10-17

**Authors:** Mathew Clement, James A. Pearson, Stephanie Gras, Hugo A. van den Berg, Anya Lissina, Sian Llewellyn-Lacey, Mark D. Willis, Tamsin Dockree, James E. McLaren, Julia Ekeruche-Makinde, Emma Gostick, Neil P. Robertson, Jamie Rossjohn, Scott R. Burrows, David A. Price, F. Susan Wong, Mark Peakman, Ania Skowera, Linda Wooldridge

**Affiliations:** 1 Division of Infection and Immunity, Cardiff University, Cardiff CF14 4XN, UK; 2 Infection and Immunity Program and Department of Biochemistry and Molecular Biology, Biomedicine Discovery Institute, Monash University, Clayton, Victoria 3800, Australia.; 3 Australian Research Council Centre of Excellence for Advanced Molecular Imaging, Monash University, Clayton, VIC 3800, Australia; 4 Mathematics Institute, University of Warwick, Coventry CV4 7AL, UK; 5 Faculty of Health Sciences, University of Bristol, Bristol BS8 1TD, UK; 6 Division of Psychological Medicine and Clinical Neuroscience, Cardiff University, Cardiff CF14 4XN, UK; 7 Mucosal Infection and Immunity Group, Department of Medicine, Imperial College London, London SW7 2AZ, UK; 8 QIMR Berghofer Medical Research Institute, Brisbane, QLD 4029, Australia; 9 Vaccine Research Center, National Institute of Allergy and Infectious Diseases, National Institutes of Health, Bethesda, MD 20892, USA; 10 Department of Immunobiology, King’s College London, London SE1 9RT, UK

## Abstract

CD8^+^ T-cells play a role in the pathogenesis of autoimmune diseases such as multiple sclerosis and type 1 diabetes. However, drugs that target the entire CD8^+^ T-cell population are not desirable because the associated lack of specificity can lead to unwanted consequences, most notably an enhanced susceptibility to infection. Here, we show that autoreactive CD8^+^ T-cells are highly dependent on CD8 for ligand-induced activation via the T-cell receptor (TCR). In contrast, pathogen-specific CD8^+^ T-cells are relatively CD8-independent. These generic differences relate to an intrinsic dichotomy that segregates self-derived and exogenous antigen-specific TCRs according to the monomeric interaction affinity with cognate peptide-major histocompatibility complex class I (pMHCI). As a consequence, “blocking” anti-CD8 antibodies can suppress autoreactive CD8^+^ T-cell activation in a relatively selective manner. These findings provide a rational basis for the development and *in vivo* assessment of novel therapeutic strategies that preferentially target disease-relevant autoimmune responses within the CD8^+^ T-cell compartment.

CD8^+^ T-cells recognise cognate peptide-major histocompatibility complex class I (pMHCI) antigens via the clonotypically-expressed αβ T-cell receptor (TCR) and the lineage-specific CD8 coreceptor^1^,^2^. The TCR engages the α1/α2 domain peptide-binding platform of pMHCI, thereby dictating antigen specificity^3^. In contrast, CD8 binds at a spatially distinct and largely conserved site formed by the α3 domain of the MHCI heavy chain with a contribution from β2-microglobulin, an event that acts functionally to enhance antigen sensitivity^4^,^5^. Several mechanisms are involved in this latter phenomenon, including: (i) stabilisation of the TCR/pMHCI interaction^6^,^7^; (ii) recruitment of essential signalling molecules to the intracellular side of the TCR/CD3/ζ complex^8^,^9^,^10^,^11^; and (iii) localisation of the TCR/pMHCI complex within membrane micro-domains that form privileged sites for the initiation of TCR-mediated signalling^12^,^13^. This allows the CD8 coreceptor to fine-tune antigen-specific responses within the CD8^+^ T-cell compartment.

It has become increasingly evident in recent years that CD8^+^ T-cells play a key role in the pathogenesis of autoimmune diseases such as type 1 diabetes (T1D)^14^,^15^,^16^ and multiple sclerosis (MS)^17^,^18^,^19^. As such, there is a strong rationale for developing therapeutic strategies that target the autoreactive CD8^+^ T-cell population^20^,^21^,^22^. Previous studies have employed antibodies directed against T-cell surface markers (CD3, CD4 and CD8) to induce tolerance in mice^20^,^21^,^23^,^24^, although to date, it has not been possible to translate strategies using “tolerance-inducing antibodies” into humans^25^. However, there are important biological differences between autoreactive and pathogen-specific CD8^+^ T-cells that may be amenable to therapeutic exploitation. Extensive biophysical analyses have shown that pathogen-specific TCRs typically engage cognate pMHCI with high monomeric affinities (range K_D_ ~1–50 μM)^3^,^26^. In contrast, autoreactive TCRs that escape negative selection display markedly lower monomeric affinities for pMHCI (K_D_ >100 μM)^27^,^28^,^29^,^30^. Autoimmune disease-relevant TCR/pMHCI interactions may even occur at K_D_ values >200 μM^31^,^32^. Importantly, CD8^+^ T-cells bearing such low affinity TCRs are highly dependent on CD8 for cognate ligand-induced activation^33^,^34^. On the basis of these observations, we hypothesised that CD8-targeted strategies could be used to inhibit autoreactive CD8^+^ T-cells in a relatively selective manner.

Anti-CD8 monoclonal antibodies have been used widely to study the functional role of the CD8 coreceptor^35^,^36^. To phenotype such antibodies we have defined the following criteria: (1) effect on pMHCI tetramer staining, (2) effect on pMHCI specific activation; and, (3) ability to trigger non-specific activation (i.e. to elicit effector function in the absence of TCR/pMHCI engagement)^37^. We have observed that considerable heterogeneity exists between different anti-CD8 antibodies. In general, anti-CD8 antibodies can inhibit or enhance pMHCI tetramer binding, which is mirrored by their effect on pMHCI antigen-specific activation^35^. It is notable that the anti-mouse CD8 antibody YTS105.18 has been used in previous studies to reverse T1D in non-obese diabetic (NOD) mice^20^,^21^. This clone does not inhibit pMHCI binding or antigen-specific CD8^+^ T-cell activation and therefore cannot be classified as a “blocking” antibody^38^. In contrast, we have selected an anti-human CD8 antibody (DK25) that exhibits a potent blocking phenotype. DK25 inhibits pMHCI tetramer binding at the cell surface, inhibits pMHCI antigen specific T-cell activation but does not trigger non-specific activation^35^,^37^.

We show that autoreactive CD8^+^ T-cells are preferentially inhibited by blocking anti-CD8 antibodies as a consequence of low affinity TCR/pMHCI interactions that confer an intrinsic dependence on the CD8 coreceptor for ligand-induced activation via the TCR. Our findings suggest novel strategies for the treatment of autoimmune diseases without the attendant side effects that complicate generalised immunosuppression.

## Results

### Autoreactive CD8^+^ T-cells expressing low affinity TCRs are highly CD8-dependent

**Figure 1:**
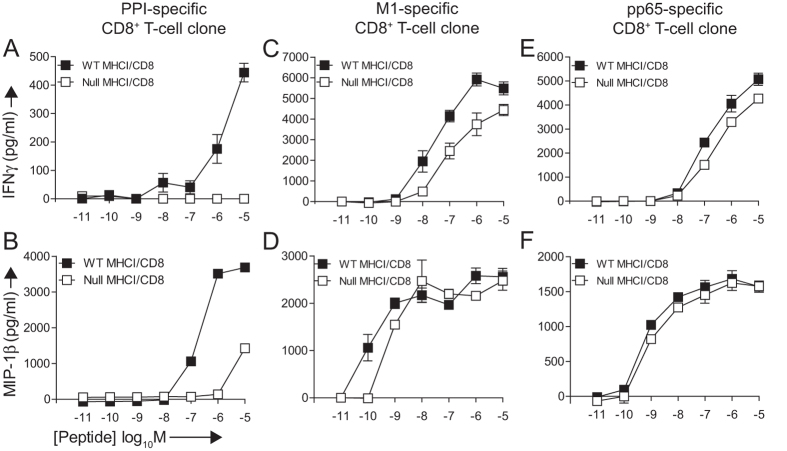
Autoreactive CD8^+^ T-cells expressing low affinity TCRs are highly CD8-dependent. 6 × 10^4^ C1R-A*0201 (WT MHCI/CD8) or C1R-A*0201 D227K/T228A (Null MHCI/CD8) target cells were pulsed with cognate peptide at the indicated concentrations in duplicate for 1 hour at 37 °C. 3 × 10^4^ clonal 1E6 (**A,B**), ALF8 (**C,D**) or MCNLV (**E,F**) CD8^+^ T-cells were then added and the plates were incubated for 4 hours at 37 °C. Supernatants were assayed for IFN-γ and MIP-1β by ELISA. The mean ± SD of two replicate assays is shown. Data is representative of three independent experiments.

A primary aim of this study was to test the hypothesis that autoreactive CD8^+^ T-cells are highly dependent on CD8 for ligand-induced activation via the TCR. In preliminary experiments, we made use of the well-characterised CD8^+^ T-cell clone 1E6, which is specific for the HLA-A*0201-restricted preproinsulin (PPI) epitope ALWGPDPAAA (**ALW**)^39^. Despite a low affinity monomeric interaction between the 1E6 TCR and cognate pMHCI (K_D_ = 278 μM), this clone recognises and destroys human pancreatic β-cells *in vitro*^32^,^39^. To examine the CD8 dependency of 1E6, we measured effector outputs in response to titrated doses of the cognate peptide ligand presented by C1R-A*0201 or C1R-A*0201 D227K/T228A cells. In the absence of pMHCI/CD8 engagement due to the HLA-A*0201 D227K/T228A mutation, interferon (IFN)-γ production by the 1E6 CD8^+^ T-cell clone was completely abrogated (Fig. 1A). Macrophage inflammatory protein (MIP)-1β release was similarly compromised at physiologically relevant peptide concentrations (Fig. 1B).

Next, we performed similar experiments with the CD8^+^ T-cell clone ALF8, which recognises the HLA-A*0201-restricted influenza A virus matrix protein (M1) epitope GILGFVFTL. The ALF8 TCR engages cognate pMHCI with a monomeric affinity (K_D_ = 7.71 μM; Table 1 and Supplementary Fig. S1) that falls within the typical range for pathogen-specific TCRs (K_D_ = 1–50 μM)^3^,^26^. ALF8 exhibited functional responses at substantially lower concentrations of exogenous peptide compared with 1E6. In the absence of an intact pMHCI/CD8 interaction, ALF8 was still capable of efficient production of IFN-γ and MIP-1β, even at low peptide concentrations (Fig. 1C,D). Similar results were obtained with the CD8^+^ T-cell clone MCNLV, which is specific for the HLA-A*0201-restricted human cytomegalovirus (HCMV) pp65 epitope NLVPMVATV (Fig. 1E,F). These data suggest that autoreactive CD8^+^ T-cells expressing low affinity TCRs are less antigen sensitive and more dependent on CD8 coreceptor engagement as compared to pathogen-specific CD8^+^ T-cells.

### CD8 has a profound effect on the number of peptides that can be recognised by autoreactive CD8^+^ T-cells

**Figure 2:**
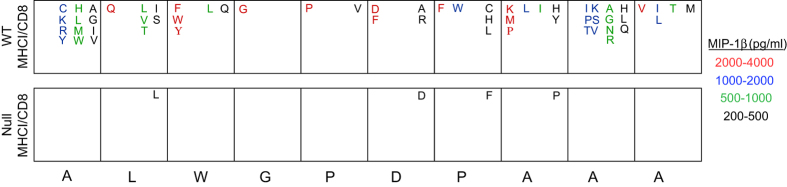
CD8 has a profound effect on the number of peptides that can be recognised by the diabetogenic CD8^+^ T-cell clone 1E6. 6 × 10^4^ C1R-A*0201 (WT MHCI/CD8) or C1R-A*0201 D227K/T228A (Null MHCI/CD8) target cells were pulsed in duplicate with each mixture from a 10mer combinatorial peptide library (100 μM) for 2 hours at 37 °C. 3 × 10^4^ clonal 1E6 CD8^+^ T-cells were then added and the plates were incubated overnight at 37 °C. Supernatants were assayed for MIP-1β by ELISA. The primary screens with wildtype (WT) targets were reported previously^40^; the primary screens with D227K/T228A (Null) targets are shown in Supplementary Fig. S2. A box plot summary is presented with the index sequence at the bottom. Data is representative of two independent experiments.

In an earlier study, we showed that the 1E6 clone is highly cross-reactive in the context of HLA-A*0201, recognising ~1 million different peptide ligands at physiologically relevant concentrations^40^. As part of this earlier study we performed a 10mer combinatorial peptide library (CPL) scan of the 1E6 clone using C1R-A*0201 targets (WT MHCI/CD8) to present each peptide mixture^40^. To extend these findings, we performed a 10mer combinatorial peptide library (CPL) scan of 1E6 using C1R-A*0201 D227K/T228A targets (Null MHCI/CD8) (Fig. 2 and Supplementary Fig. S2). The 1E6 clone is highly promiscuous in the presence of an intact pMHCI/CD8 interaction (Fig. 2). However, in the absence of CD8 engagement, very few responses were observed (Fig. 2 and Supplementary Fig. S2). This is consistent with a previous study that demonstrates that CD8 can control levels of T-cell crossreactivity^34^. Abrogation of the pMHCI/CD8 interaction eliminates promiscuous pMHCI recognition of the 1E6 TCR.

### Selection of a potent anti-CD8 antibody that blocks pMHCI tetramer binding at low TCR/pMHCI affinities

**Figure 3:**
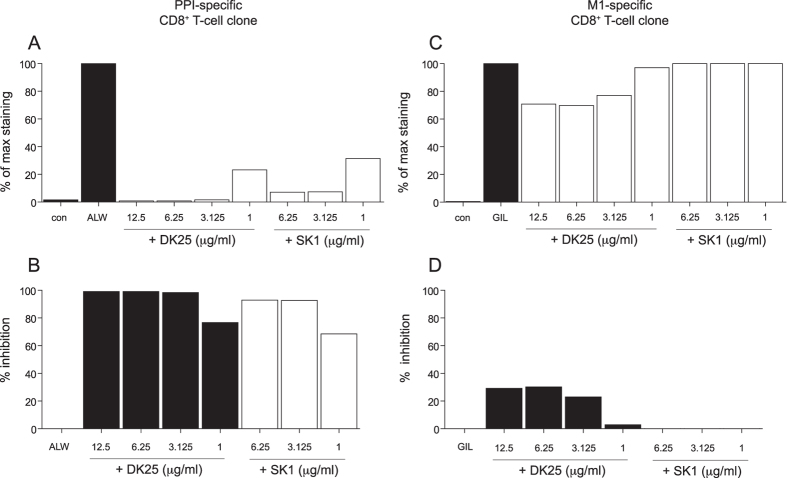
Anti-CD8 antibodies can block pMHCI tetramer binding at low TCR/pMHCI affinities. 5 × 10^4^ clonal 1E6 (**A,B**) or ALF8 (**C,D**) CD8^+^ T-cells were pre-treated with or without the indicated anti-human CD8 antibodies at various concentrations for 20 minutes at 4 °C and then stained with 25 μg/ml of cognate pMHCI tetramer for 15 minutes at 37 °C. The column graphs depict percent maximal tetramer staining (**A,C**) or percent inhibition of tetramer staining (**B,D**). Data is representative of three independent experiments.

It is established that anti-CD8 antibodies display various phenotypes^35^,^37^. In particular, SK1 and DK25 can be classified as blocking anti-CD8 antibodies because they inhibit pMHCI binding at the cell surface. Consistent with this trait, both SK1 and DK25 blocked pMHCI tetramer staining of the 1E6 clone in a concentration-dependent manner (Fig. 3A,B). In contrast, pMHCI tetramer staining of the ALF8 clone was minimally disrupted by the same antibodies across an identical concentration range (Fig. 3C,D). These results suggest that anti-CD8 antibody-mediated blockade of pMHCI binding occurs selectively in the presence of a weak TCR/pMHCI interaction. Further experiments designed to extend this observation were conducted with DK25, which exhibited more potent blocking activity in side-by-side comparisons with SK1 (Fig. 3A–D).

### DK25 blocks the activation of CD8^+^ T-cells at low TCR/pMHCI affinities

**Figure 4:**
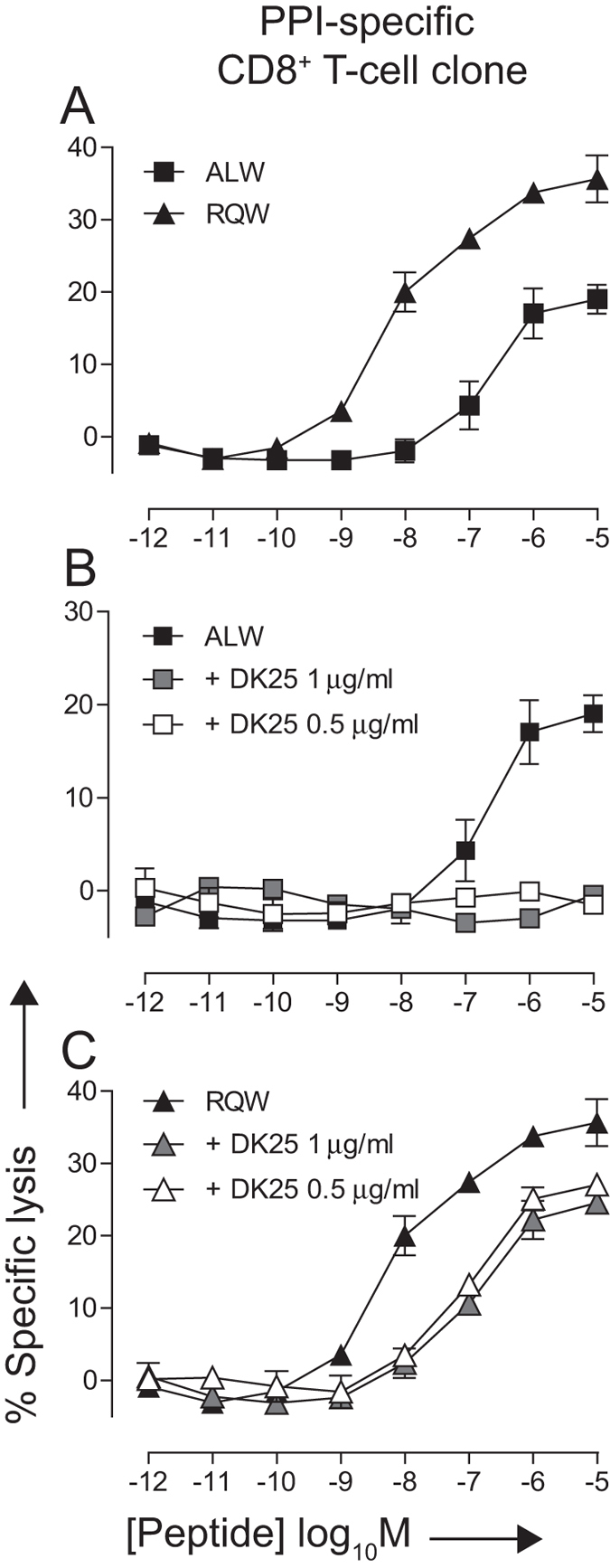
DK25 blocks the activation of CD8^+^ T-cells at low TCR/pMHCI affinities. 2 × 10^3 51^Cr-labeled C1R-A*0201 target cells were peptide-pulsed with either ALWGPDPAAA (**A,B**) or RQWGPDPAAV (**A,C**) at the indicated concentrations for 1 hour at 37 °C. 1 × 10^4^ clonal 1E6 CD8^+^ T-cells were then added with or without the anti-human CD8 antibody DK25 at a final concentration of 0.5 or 1 μg/ml. Cytotoxicity was determined after 4 hours as described in the Materials and Methods. The mean ± SD of three replicate assays is shown. Data is representative of three independent experiments.

To test the hypothesis that DK25 can be used as a selective agent to target CD8^+^ T-cells bearing low affinity TCRs, we again employed the 1E6 clone and examined activation in response to both the cognate ligand (**ALW**) and the altered peptide variant **RQ**WGPDPAA**V** (**RQW**), which binds the 1E6 TCR with a substantially higher monomeric affinity (K_D_ = 10 μM)^41^. This monoclonal system allowed a rigorous evaluation of DK25-mediated effects as a function of a single variable parameter, namely the affinity of the TCR/pMHCI interaction. In cytotoxicity assays, the 1E6 clone killed target cells presenting either **ALW** or **RQW** at levels proportional to the corresponding TCR/pMHCI affinities (Fig. 4A). This is consistent with a recent publication that demonstrates a strong correlation between 1E6 T-cell antigen sensitivity and TCR/pMHCI affinity^42^. Interestingly, there is no correlation between ligand potency and pMHCI stability in this system (T_m_ for A2-ALW and A2-RQW was 60 °C and 54 °C, respectively)^42^. The addition of DK25 at low concentrations (0.5 or 1 μg/ml) abolished the killing response to target cells presenting **ALW** (Fig. 4B). In contrast, the same concentrations of DK25 failed to abolish 1E6-mediated killing of target cells presenting **RQW** (Fig. 4C). These data suggest that DK25 selectively inhibits CD8^+^ T-cell activation in the presence of low affinity TCR/pMHCI interactions.

### DK25 selectively blocks autoreactive CD8^+^ T-cell activation

**Figure 5:**
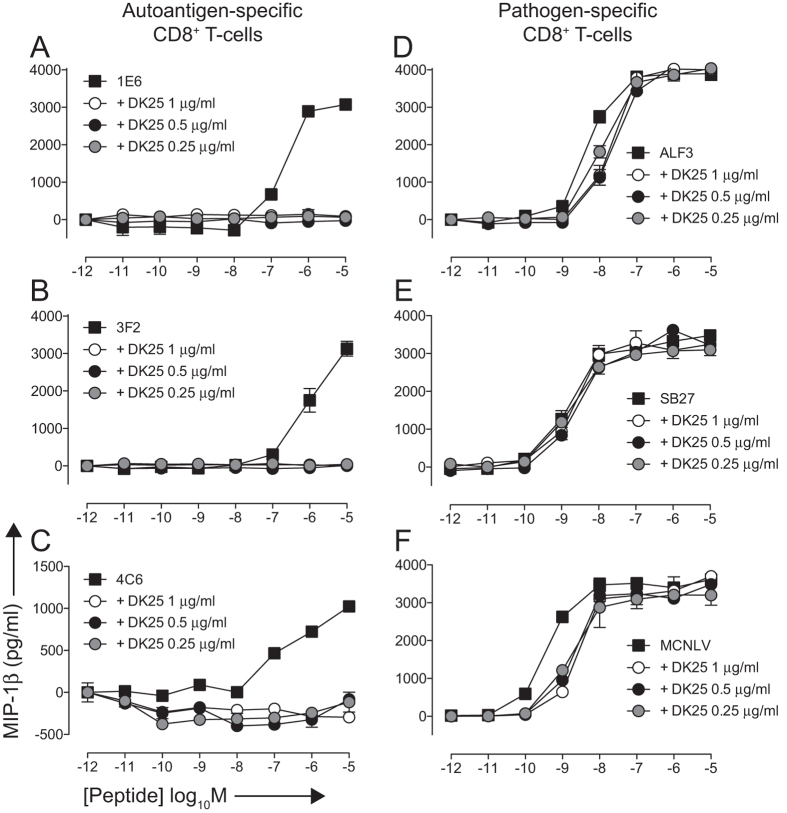
DK25 selectively blocks autoreactive CD8^+^ T-cell activation. 6 × 10^4^ C1R-A*0201 target cells were peptide-pulsed with ALWGPDPAAA (**A**,**B**), GILGFVFTL (**D**) or NLVPMVATV (**F**) at the indicated concentrations for 1 hour at 37 °C. In parallel, 6 × 10^4^ K562-A*2402 or C1R-B*3508 target cells were peptide-pulsed similarly with LWMRLLPLL (**C**) or LPEPLPQGQLTAY (**E**), respectively. 3 × 10^4^ clonal 1E6 (**A**), 3F2 (**B**), 4C6 (**C**), ALF3 (**D**), SB27 (**E**) or MCNLV (**F**) CD8^+^ T-cells pre-treated for 30 minutes at 37 °C with the anti-human CD8 antibody DK25 at the indicated concentrations were then added and the plates were incubated for 4 hours at 37 °C. Supernatants were assayed for MIP-1β by ELISA. The mean ± SD of two replicate assays is shown. Data is representative of two independent experiments.

Next, we evaluated the ability of DK25 to inhibit autoreactive and pathogen-specific CD8^+^ T-cell clones in parallel activation assays. Complete blockade of MIP-1β release by the 1E6 clone in response to the cognate peptide **ALW** was observed in the presence of DK25 at all concentrations tested (0.25, 0.5 and 1 μg/ml; Fig. 5A). In contrast, the same concentrations of DK25 minimally impacted the activation of ALF3, a CD8^+^ T-cell clone specific for the HLA-A*0201-restricted influenza A virus M1 epitope GILGFVFTL (Fig. 5D). Consistent with this observation, the ALF3 TCR engaged cognate pMHCI with a high monomeric affinity (K_D_ = 5.84 μM; Table 1 and Supplementary Fig. S1).

**Figure 6:**
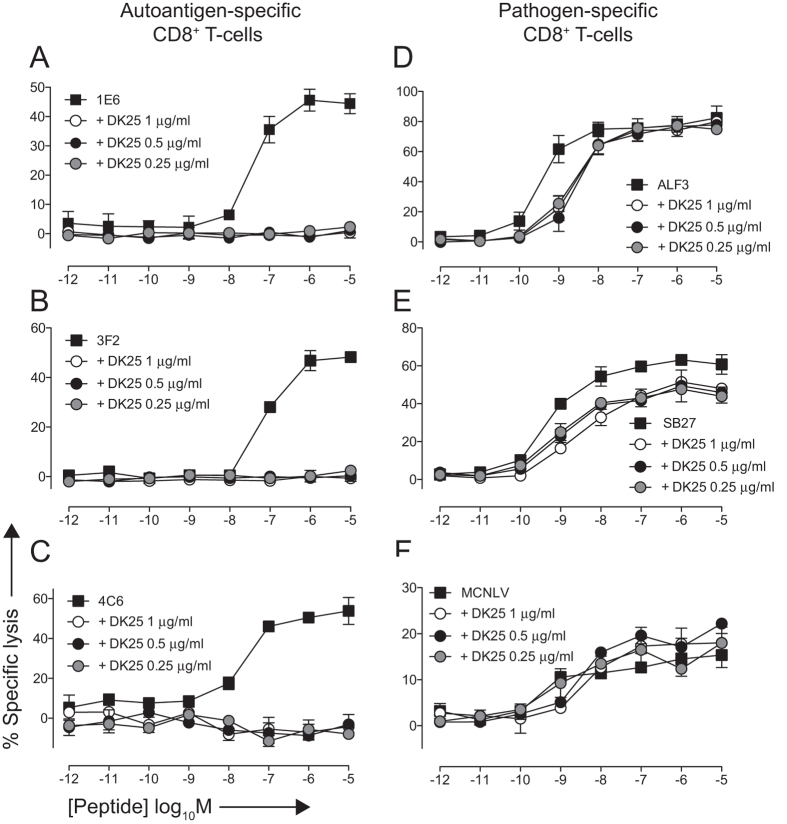
DK25 selectively blocks autoreactive CD8^+^ T-cell killing. 2 × 10^3 51^Cr-labeled C1R-A*0201 (**A,B,D,F**), K562-A*2402 (**C**) or C1R-B*3508 (**E**) target cells were peptide-pulsed with ALWGPDPAAA (**A,B**), LWMRLLPLL (**C**), GILGFVFTL (**D**), LPEPLPQGQLTAY (**E**) or NLVPMVATV (**F**) at the indicated concentrations for 1 hour at 37 °C. 1 × 10^4^ clonal 1E6 (**A**), 3F2 (**B**), 4C6 (**C**), ALF3 (**D**), SB27 (**E**) or MCNLV (**F**) CD8^+^ T-cells were then added with or without the anti-human CD8 antibody DK25 at a final concentration of 0.25, 0.5 or 1 μg/ml. Cytotoxicity was determined after 4 hours as described in the Materials and Methods. The mean ± SD of three replicate assays is shown and is representative of two independent experiments.

We conducted similar experiments across a range of different epitope specificities and HLA class I restriction elements. The autoreactive CD8^+^ T-cell clones 3F2 and 4C6 recognise the HLA-A*0201-restricted PPI epitope **ALW** and the HLA-A*2402-restricted PPI epitope LWMRLLPLL, respectively. In both cases, MIP-1β release was completely inhibited by DK25 across all cognate peptide concentrations tested (Fig. 5B,C). The pathogen-specific CD8^+^ T-cell clones SB27 and MCNLV recognise the HLA-B*3508-restricted Epstein-Barr virus (EBV) BZLF1 epitope LPEPLPQGQLTAY and the HLA-A*0201-restricted HCMV pp65 epitope NLVPMVATV, respectively. In both cases, robust peptide-specific activation was observed in the presence of DK25 (Fig. 5E,F). The SB27 TCR has been well characterised previously and binds cognate pMHCI with a high monomeric affinity (K_D_ = 9.9 μM)^43^,^44^. Comparable results were obtained for all clones in cytotoxicity assays (Fig. 6A–F). These differences in susceptibility to inhibition by DK25, comparing grouped autoreactive versus pathogen-specific CD8^+^ T-cells, were significant at the 5% level (Wilcoxon-Mann-Whitney rank sum test). Anti-CD8 antibodies can therefore be used to block the activation of autoreactive CD8^+^ T-cells in a selective manner regardless of epitope specificity or HLA class I restriction.

### DK25 inhibits pancreatic β-cell killing by PPI-specific CD8^+^ T-cells

**Figure 7:**
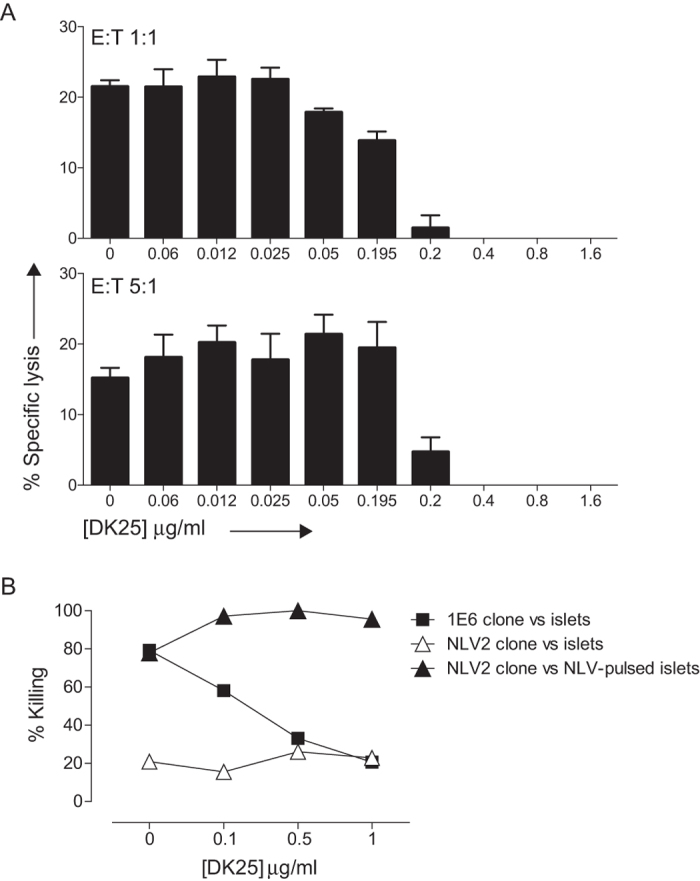
DK25 inhibits pancreatic β-cell killing by autoreactive CD8^+^ T-cells. (**A**) 2 × 10^3 51^Cr-labeled K562-PPI-A*0201 cells were peptide-pulsed with ALWGPDPAAA at a concentration of 10^−5^ M for 1 hour at 37 °C. Clonal 1E6 CD8^+^ T-cells were then added at an E:T ratio of 1:1 (upper panel) or 5:1 (lower panel) with or without the anti-human CD8 antibody DK25 at the indicated concentrations. Cytotoxicity was determined after 4 hours as described in the Materials and Methods. The mean ± SD of three replicate assays is shown and is representative of three independent experiments. (**B**) 5 × 10^3^ HLA-A*0201^+^ islet cells were incubated for 4 hours with clonal 1E6 or NLV2 CD8^+^ T-cells pre-treated for 30 minutes at 37 °C with the anti-human CD8 antibody DK25 at the indicated concentrations. As a control, islet cells were peptide-pulsed with NLVPMVATV at a concentration of 5 μg/ml for 30 minutes at 37 °C. All data points represent the mean of triplicate evaluations at a final E:T ratio of 25:1. Islet cell killing was measured using a non-radioactive europium hydrophilic ligand assay as described in the Materials and Methods. Significance of the effect of DK25 was evaluated using linear regression and the t-test statistic as described in the materials and methods.

To examine the utility of anti-CD8 antibody-mediated blockade in a more physiological setting, we tested the ability of DK25 to inhibit pancreatic β-cell killing by the 1E6 clone. In preliminary experiments, we made use of the surrogate β-cell line K562-PPI-A*0201, created by transfecting K562 leukemia cells with PPI and HLA-A*0201. DK25 blocked 1E6-mediated killing of K562-PPI-A*0201 cells at concentrations >0.2 μg/ml (Fig. 7A), consistent with the exogenous peptide titration data (Figs 5 and 6). We then performed a similar experiment using pancreatic β-cells from an HLA-A*0201^+^ donor (Fig. 7B). In the absence of DK25, pancreatic β-cells were readily killed by 1E6. The CD8^+^ T-cell clone NLV2, which was derived from a T1D patient as a control and recognises the HLA-A*0201-restricted HCMV pp65 epitope NLVPMVATV, displayed no direct lytic activity in the same assay. Pancreatic β-cell killing by 1E6 was inhibited by DK25 in a dose-dependent manner, reaching background levels at antibody concentrations ≥0.5 μg/ml. In contrast, DK25 had no effect on the ability of NLV2 to kill cognate peptide-pulsed HLA-A*0201^+^ pancreatic β-cells. There was a highly significant dependence on DK25 concentration for 1E6 killing of pancreatic β-cells (P-value = 0.0078), whereas the dependence on DK25 concentration for NLV2 was non-significant (NLV2 vs pancreatic β-cells: P = 0.52 and NLV2 vs NLV-pulsed pancreatic β-cells: P = 0.28). Therefore, relatively low concentrations of DK25 can therefore be used to inhibit CD8^+^ T-cell-mediated pancreatic β-cell destruction without impacting pathogen-specific immunity.

### DK25 efficiently blocks the activation of myelin-specific CD8^+^ T-cells

**Figure 8:**
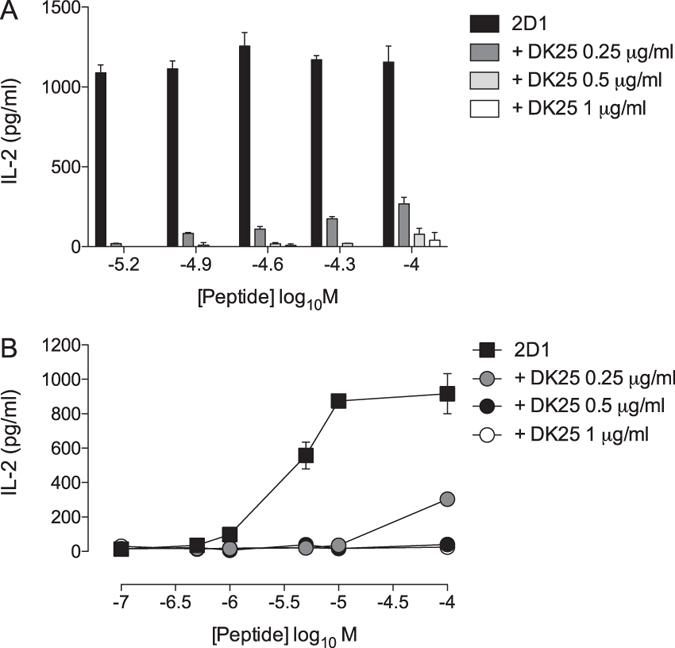
DK25 inhibits the activation of myelin-specific CD8^+^ T-cells. 3 × 10^4^ BW58 cells expressing human CD8αβ and the 2D1 TCR were stimulated overnight at 37 °C in the presence of 6 × 10^4^ KLIETYFSK peptide-pulsed HLA-A*0301^+^ B-LCL target cells with or without the anti-human CD8 antibody DK25 at a final concentration of 0.25, 0.5 or 1 μg/ml (**A,B**). Supernatants were assayed for IL-2 by ELISA. The mean ± SD of two replicate assays is shown in (**B**) and is representative of three independent experiments.

To extend our study to other autoimmune targets, we examined the effect of DK25 on the activation of BW58 cells expressing human CD8αβ and the 2D1 TCR, which is specific for the HLA-A*0301-restricted myelin proteolipid protein (PLP) epitope KLIETYFSK. The 2D1 TCR plays a role in the induction of a multiple sclerosis-like disease in humanised mice expressing HLA-A*0301^45^. DK25 efficiently blocked peptide-induced interleukin (IL)-2 production by the BW58 cell line at concentrations of 0.5 and 1 μg/ml (Fig. 8A,B). Incomplete but nonetheless potent suppression was observed at an antibody concentration of 0.25 μg/ml. Anti-CD8 antibody-mediated blockade of autoreactive CD8^+^ T-cells is therefore not restricted by tissue specificity.

### Blocking anti-mouse CD8 antibodies inhibit autoreactive CD8^+^ T-cell activation

**Figure 9:**
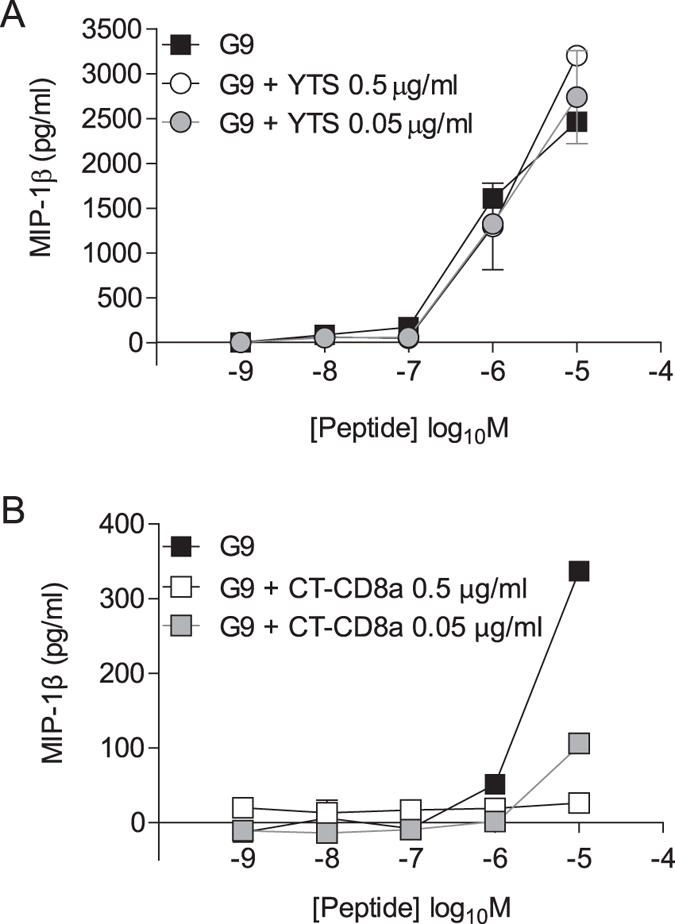
CT-CD8a blocks the activation of autoreactive murine CD8^+^ T-cells. 6 × 10^4^ P815 murine mastocytoma cells were peptide-pulsed with LYLVCGERG at the indicated concentrations for 1 hour at 37 °C. 3 × 10^4^ splenic G9C8 TCR-transgenic CD8^+^ T-cells pre-treated for 30 minutes at 37 °C with either the anti-mouse CD8 antibody YTS105.18 (**A**) or the anti-mouse CD8 antibody CT-CD8a (**B**) at the indicated concentrations were then added and the plates were incubated overnight at 37 °C. Supernatants were assayed for MIP-1β by ELISA. The mean ± SD of two replicate assays is shown and is representative of two independent experiments.

Two previous studies have used anti-CD8 antibodies to prevent and reverse TID in mice^20^,^21^. In both cases, the investigators relied principally on the anti-mouse CD8 antibody YTS105.18. Shore *et al*. demonstrated that YTS105.18 does not block pMHCI tetramer binding or CD8^+^ T-cell activation^38^. We confirmed this observation by showing that murine CD8^+^ T-cells expressing the G9C8 TCR, which is specific for the H-2K^d^-restricted insulin B chain epitope LYLVCGERG, exhibit robust activation in the presence of YTS105.18 (Fig. 9A). The blocking approach presented in this study is therefore distinct from the phenomena described in these earlier reports.

Next, we tested the anti-mouse CD8 antibody CT-CD8a, which inhibits pMHCI tetramer binding and CD8^+^ T-cell activation^35^,^37^. The G9C8 TCR binds cognate pMHCI with a low monomeric affinity (K_D_ = 286 μM)^46^. In line with our observations in human systems, CT-CD8a efficiently blocked peptide-induced activation of G9C8-expressing CD8^+^ T-cells (Fig. 9B). However, CT-CD8a cannot be used therapeutically *in vivo* because it triggers non-specific CD8^+^ T-cell activation with exposure times ≥18 hours^37^. To date, it has not been possible to identify a blocking anti-mouse CD8 antibody with a phenotype akin to DK25, which may be due to intrinsic biophysical and structural differences between mice and humans with respect to the pMHCI/CD8 interaction^47^.

## Discussion

Over the past decade, a substantial body of evidence has accumulated to implicate CD8^+^ T-cells as key players in the pathogenesis of common autoimmune diseases such as T1D^14^,^15^,^16^,^48^,^49^, MS^17^,^20^,^50^ and psoriasis^51^. Autoreactive TCRs typically engage cognate pMHCI with low monomeric affinities (K_D_s >100 μM), in contrast to pathogen-specific TCRs (K_D_s ~1–50 μM)^26^. It is also established that CD8^+^ T-cells expressing low affinity TCRs are strictly dependent on CD8 for ligand-induced activation^33^,^34^. These observations led us to hypothesise that CD8-targeted strategies could be used to block CD8^+^ T-cell-mediated autoreactivity in a selective manner.

In preliminary experiments, we tested this possibility using the well-characterised autoreactive CD8^+^ T-cell clone 1E6, which was isolated from a patient with T1D and displays potent reactivity against pancreatic β-cells^39^. The cognate epitope **ALW** is naturally processed from PPI and represents a major target for circulating effector CD8^+^ T-cells in HLA-A*0201^+^ patients with TID^39^. We found that peptide-induced activation of 1E6 is highly dependent on CD8, consistent with the previously reported low affinity TCR/pMHCI interaction (K_D_ = 278 μM)^31^,^32^. It is also established that 1E6 is extremely cross-reactive in the context of HLA-A*0201, recognising ~1 million different peptide ligands at physiologically relevant concentrations^40^. In line with the strict requirement for CD8, we observed a substantial winnowing of ligand cross-recognition in the absence of an intact pMHCI/CD8 interaction. Based on these findings and the fact that pathogen-specific CD8^+^ T-cells displayed higher levels of functional sensitivity and robust activation profiles irrespective of CD8 engagement, we explored the potential utility of CD8-specific antibodies as a means to block CD8^+^ T-cell activation selectively in response to self-derived peptides.

Previous work has shown that anti-CD8 antibodies exhibit diverse functional and phenotypic characteristics^35^,^37^. We selected the antibody clones SK1 and DK25 for further evaluation in view of the fact that they inhibit pMHCI tetramer binding and CD8^+^ T-cell activation in the absence of non-specific stimulatory effects. The blocking effect on pMHCI tetramer binding was concentration-dependent in both cases and only apparent at low TCR/pMHCI affinities. The DK25 clone displayed greater efficacy in this regard and was therefore used in subsequent experiments.

To test the ability of DK25 to inhibit CD8^+^ T-cell activation as a function of TCR/pMHCI affinity, we utilised a monoclonal system in which an HLA-A*0201-restricted altered peptide ligand was used to generate a high affinity interaction with the clonotypically-expressed 1E6 TCR. DK25 efficiently blocked activation of the 1E6 clone in response to the cognate ligand **ALW**, whereas only a marginal effect was observed with the altered peptide ligand **RQW**, which resembles a pathogen-derived epitope in terms of TCR/pMHCI affinity (K_D_ = 10 μM)^3^,^41^. Similar results were obtained across a panel of different autoreactive (1E6, 3F2 and 4C6) and pathogen-specific (ALF8, SB27 and MCNLV) CD8^+^ T-cell clones. These findings suggest that blocking anti-CD8 antibodies can be used as selective agents for therapeutic purposes in the setting of autoimmune disease. In support of this notion, we found that DK25 can prevent CD8^+^ T-cell-mediated destruction of pancreatic β-cells and block the activation of myelin-specific CD8^+^ T-cells. As such, further research is warranted into the utility of this approach in the treatment of CD8^+^ T-cell mediated autoimmunity. Although, it is important to note that more work is required to identify pMHCI antigens important in these diseases and assess the affinity of the TCRs that recognise them to understand the scope for this approach.

Previous studies have examined the ability of anti-CD8 antibodies to induce tolerance in murine models of TID^20^,^21^. However, the YTS105.18 antibody clone used for this purpose does not inhibit either pMHCI tetramer binding or peptide-induced CD8^+^ T-cell activation^38^. The tolerising effect of YTS105.18 is therefore distinct from the novel blocking strategy proposed here. In contrast, the CT-CD8a antibody displays an inhibitory phenotype in mice akin to SK1 and DK25 in humans^35^. Autoreactive murine CD8^+^ T-cells were highly susceptible to CT-CD8a-mediated blockade *in vitro*, but the presence of non-specific stimulatory effects after prolonged exposure precluded confirmatory experiments *in vivo*^37^.

The next step will be to identify a suitable *in vivo* model to test the utility of this approach, to establish parameters such as optimal antibody concentration and assess the effect of targeting CD8 on the ability to mount an adequate pathogen specific response. The current literature suggests that during an acute response to pathogen, a broad range of TCR affinities are mobilised^52^. However, strong TCR ligation is required to sustain a T-cell response and as such, the TCR repertoire “matures” so that predominantly higher affinity TCRs remain^52^. Therefore, inhibiting the activation of low affinity pathogen-specific T-cells would not be anticipated to have a major impact on the ability to control infection as there would exist a degree of redundancy in the mobilised TCR repertoire, such that higher affinity TCRs would still be able to expand and control infection. Future *in vivo* work would also allow us to assess the possibility that autoreactive CD8^+^ T-cells with higher than expected TCR affinities (K_D_s <100 μM) occur in the repertoire and the impact that this may have on the effectiveness of this approach.

The DK25 antibody targets CD8α, which is expressed not only as a heterodimer with CD8β, exclusively on CD8^+^ T-cells, but also in homodimeric form on dendritic cells, natural killer cells and γδ T-cells. Although the precise role of CD8αα is unclear, it is thought to act as a modulator of immune function^53^,^54^. The impact of chain specificity as a determinant of *in vivo* effects will therefore require further investigation as it is anticipated that antibodies which exclusively target the CD8αβ heterodimer would be more desirable and associated with a more predictable outcome. The exact mechanism of anti-CD8 antibody action is still unclear^35^,^37^. In addition, the use of monoclonal antibodies as therapeutics is often not ideal because they can be costly to produce, can mediate side effects (via FcR binding) and require intravenous administration. Although F(ab’)2 fragments retain the same phenotype as the parental antibody, the overall magnitude of the affect can be reduced^37^. Therefore, a viable alternative would be to design small molecular inhibitors that block the interaction between pMHCI and CD8β.

Current treatments for autoimmune and inflammatory diseases often rely on the induction of profound and/or long-lasting immunosuppression^55^,^56^,^57^. However, adverse effects are common and often relate to a negative impact on protective immune responses. Our data suggest that autoreactive CD8^+^ T-cells can be preferentially targeted using blocking anti-CD8 antibodies. Additional modalities could also be employed based on the underlying biology, such as rationally designed small molecular inhibitors and soluble versions of the inhibitory immunoglobulin-like transcript 2 (ILT2) receptor^58^. These approaches hold the potential to suppress pathogenic CD8^+^ T-cell-mediated autoimmunity without the attendant side effects that complicate standard therapeutic interventions.

## Materials and Methods

### Cells

The following CD8^+^ T-cell clones were used in this study: (i) 1E6 and 3F2, specific for the HLA-A*0201-restricted PPI epitope ALWGPDPAAA (residues 15–24)^39^; (ii) ALF3 and ALF8, specific for the HLA-A*0201-restricted influenza A virus M1 epitope GILGFVFTL (residues 58–66)^37^; (iii) MCNLV and NLV2, specific for the HLA-A*0201-restricted HCMV pp65 epitope NLVPMVATV (residues 495–503); (iv) 4C6, specific for the HLA-A*2402-restricted PPI epitope LWMRLLPLL (residues 3–11)^59^; and (v) SB27, specific for the HLA-B*3508-restricted EBV BZLF1 epitope LPEPLPQGQLTAY (residues 52–64)^60^. Equilibrium binding affinities for the corresponding TCR/pMHCI interactions are shown in Table 2. C1R target cells expressing HLA-A*0201 (C1R-A*0201), HLA-A*0201 D227K/T228A (C1R-A*0201 D227K/T228A) or HLA-B*3508 (C1R-B*3508) were generated as described previously^61^. K562 cells expressing HLA-A*2402 (K562-A*2402) and BW58 cells expressing human CD8αβ and the 2D1 TCR, which is specific for the HLA-A*0301-restricted myelin PLP epitope KLIETYFSK (residues 45–53), were also described previously^39^,^45^. All CD8^+^ T-cells were maintained in RPMI 1640 containing 100 U/ml penicillin, 100 mg/ml streptomycin, 2 mM L-glutamine and 10% heat-inactivated fetal calf serum (all Life Technologies) supplemented with 2.5% Cellkines (Helvetica Healthcare), 200 IU/ml IL-2 and 25 ng/ml IL-15 (both PeproTech).

### G9 transgenic mice

Transgenic mice expressing the G9C8 TCR on the NOD.TCRCα^−/−^ genetic background were generated as described previously^62^,^63^. Mice were housed in microisolators or scantainers in the specific pathogen-free facility at Cardiff University. The G9C8 TCR is specific for the H-2K^d^-restricted insulin B chain epitope LYLVCGERG (residues 15–23)^62^,^64^. All procedures were performed in accordance with protocols approved by the UK Home Office.

### Anti-CD8 antibodies

The following antibodies were used in this study: (i) APC-conjugated anti-human CD8α (DK25; DAKO); (ii) purified unconjugated anti-human CD8α (SK1; BD Biosciences); (iii) purified unconjugated anti-mouse CD8α (CT-CD8a; Lifespan BioScience); and (iv) purified unconjugated anti-mouse CD8α (YTS105.18; Thermo Scientific).

### CD8^+^ T-cell effector function assays

6 × 10^4^ C1R-A*0201, C1R-A*0201 D227K/T228A, C1R-B*3508, K562-A*2402 or HLA-A*0301^+^ B-lymphoblastoid cell line (B-LCL) targets were pulsed with various concentrations of cognate peptide as indicated for 1 hour at 37 °C. 3 × 10^4^ clonal CD8^+^ T-cells pre-treated for 30 minutes at 4 °C with various concentrations of the anti-human CD8 antibody DK25 as indicated were incubated with peptide-pulsed targets for 4 hours at 37 °C. Untreated effectors and unpulsed targets were used as controls. Supernatants were harvested and assayed for IFN-γ and MIP-1β by ELISA (R&D Systems).

### Combinatorial peptide library screens

A 10mer CPL in positional scanning format was purchased from Pepscan. CPL scans were performed as described previously^40^,^65^. Briefly, 6 × 10^4^ C1R-A*0201 or C1R-A*0201 D227K/T228A cells were pulsed with each of the 10mer CPL mixtures (100 μM) in duplicate for 2 hours at 37 °C. 3 × 10^4^ clonal CD8^+^ T-cells were then added and the plates were incubated overnight at 37 °C. Supernatants were harvested and assayed for MIP-1β by ELISA (R&D Systems).

### pMHCI tetramer staining

Soluble biotinylated pMHCI proteins were manufactured and tetramerised with fluorochrome-labeled streptavidin as described previously^6^. 5 × 10^4^ clonal CD8^+^ T-cells pre-treated with or without the indicated anti-human CD8 antibodies at various concentrations in 20 μl of PBS for 20 minutes at 4 °C were stained with 25 μg/ml of cognate pMHCI tetramer for 15 minutes at 37 °C. The cells were then washed and stained with LIVE/DEAD Fixable Aqua or Violet (Life Technologies) for 10 minutes at room temperature. Data were acquired using a FACSCantoII flow cytometer (BD Biosciences) and analysed with FlowJo software (TreeStar).

### Chromium release assay

2 × 10^3^ C1R-A*0201, K562-A*2402 or C1R-B*3508 targets were labeled with 30 μCi of ^51^Cr per 10^6^ cells for 1 hour at 37 °C in RPMI 1640 containing 100 U/ml penicillin, 100 mg/ml streptomycin, 2 mM L-glutamine and 2% heat-inactivated fetal calf serum (all Life Technologies). Labeled targets were then pulsed with peptide for 1 hour at 37 °C. CD8^+^ T-cells pre-treated with or without the indicated anti-human CD8 antibodies at various concentrations for 30 minutes at 4 °C were added at an effector-to-target (E:T) ratio of 5:1 and the plates were incubated for 4 hours at 37 °C. Spontaneous release was determined using targets incubated alone. Total release was determined using targets incubated in the presence of 5% Triton X-100 (Sigma-Aldrich). For each sample, 10 μl of supernatant was mixed with 150 μl of OptiPhase Supermix Scintillation Cocktail (Perkin Elmer) and analysed using a MicroBeta TriLux Liquid Scintillation and Luminescence Counter (Perkin Elmer). Specific lysis was calculated using the following formula:


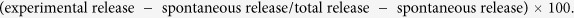


### Human islet killing assays

Human islet isolations were performed as described previously using pancreata retrieved with the consent of donors’ relatives and permission from the Ethical Review Committee of King’s College Hospital^66^. Islet cells were cultured in monolayers for 16–24 hours with medium containing 16 μmol glucose plus IL-1β (50 IU/ml; Strathmann Biotec), tumor necrosis factor (TNF)-α (2500 IU/ml; Miltenyi Biotec), IFN-γ (500 IU/ml; Miltenyi Biotec) and IFN-α (1000 IU/ml; Roche Laboratories) to increase MHCI expression^39^. Clonal CD8^+^ T-cells pre-treated for 30 minutes at 37 °C with various concentrations of the anti-human CD8 antibody DK25 as indicated were then incubated with 5 × 10^3^ islet cell targets at an E:T ratio of 25:1 for 4 hours at 37 °C. Cytotoxicity was analysed using a non-radioactive europium hydrophilic ligand assay with DELFIA Technology (Perkin Elmer)^39^,^67^.

### Protein expression, purification and surface plasmon resonance

The ALF3 and ALF8 TCRs were expressed, refolded and purified using an engineered disulfide linkage between the α and β constant domains^68^. Soluble HLA-A*0201 incorporating the influenza A virus M1 peptide GILGFVFTL was prepared as described previously^69^. All surface plasmon resonance experiments were conducted at 25 °C using a BIAcore 3000 instrument with HBS buffer (10 mM HEPES pH 7.4, 150 mM NaCl and 0.005% surfactant P20) containing 1% BSA to prevent non-specific binding. The human TCR-specific monoclonal antibody 12H8^70^, which recognises a conformation-dependent epitope, was amine-coupled to research-grade CM5 chips. Ligand binding to solid-phase TCRs was then measured over a pMHCI concentration range of 0.78–200 μM according to established protocols^68^. Data were analysed using BIAevaluation software version 3.1 with the 1:1 Langmuir binding model.

### Statistical analysis

DK25-induced shifts in functional sensitivity were quantified by calculating differences in pEC_50_ (delta pEC_50_), a parameter determined as −log [50% maximal response peptide concentration] for each condition (Supplementary Table 1)^71^. Differences in susceptibility to inhibition by DK25, comparing grouped autoreactive versus pathogen-specific CD8^+^ T-cells, were assessed using the Wilcoxon-Mann-Whitney rank sum test^72^. The effect of DK25 in pancreatic islet experiments was assessed using linear regression and the t-test statistic with built-in tools in *Mathematica*^72^.

## Additional Information

**How to cite this article**: Clement, M. *et al*. Targeted suppression of autoreactive CD8^+^ T-cell activation using blocking anti-CD8 antibodies. *Sci. Rep.* **6**, 35332; doi: 10.1038/srep35332 (2016).

## Supplementary Material

Supplementary Information

## Figures and Tables

**Table 1:** Affinities and kinetics of GILGFVFTL-HLA-A*0201 binding to the ALF3 and ALF8 TCRs.

TCR	K_Deq_ (μM)	k_on_ (10^4^/Ms)	k_off_ (1/s)	K_Dcalc_ (μM)	t_1/2_ (s)
ALF3	5.84 ± 0.10	2.19 ± 0.44	0.128 ± 0.007	7.33 ± 0.40	7.8
ALF8	7.71 ± 0.17	2.89 ± 0.29	0.158 ± 0.005	5.48 ± 0.39	6.3

K_Deq_ is the measured equilibrium affinity, k_on_ is the association rate, k_off_ is the dissociation rate, t_1/2_ is the half-life (0.693/k_off_) and K_Dcalc_ is the calculated affinity based on kinetic parameters.

**Table 2:** Measured TCR/pMHCI equilibrium binding affinities (K_Deq_) for the CD8^+^ T-cell clones used in this study.

TCR	Specificity	HLA restriction	Minimal epitope	K_Deq_ (μM)
1E6	PPI	A*0201	ALWGPDPAAA	278^31^,^32^
1E6	PPI	A*0201	RQWGPDPAAV	10^41^
3F2	PPI	A*0201	ALWGPDPAAA	>250^73^
4C6	PPI	A*2402	LWMRLLPLL	100^73^
SB27	EBV	B*3508	LPEPLPQGQLTAY	9.9^43^,^44^
