# Supplementary material for: Targeted suppression of autoreactive CD8+ T-cell activation using blocking anti-CD8 antibodies
Source: Sci Rep. 2016 Oct 17;6:35332. doi: 10.1038/srep35332 (PMC5066216; doi:10.1038/srep35332)
Supplement: Supplementary Information [file srep35332-s1.pdf]

Targeted suppression of autoreactive $CD8^+$ T-cell activation using blocking anti-CD8 antibodies

Mathew Clement, James A. Pearson, Stephanie Gras, Hugo A. van den Berg, Anya Lissina, Sian Llewellyn-Lacey, Mark D. Willis, Tamsin Dockree, James E. McLaren, Julia Ekeruche-Makinde, Emma Gostick, Neil P. Robertson, Jamie Rossjohn, Scott R. Burrows, David A. Price, F. Susan Wong, Mark Peakman, Ania Skowera, Linda Wooldridge

Figure S1

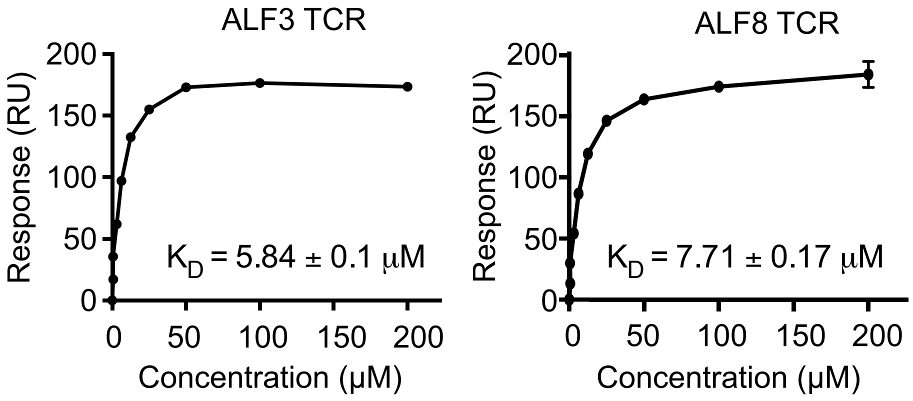

Figure S1: Affinities and kinetics of GILGFVFTL-HLA-A*0201 binding to the ALF3 and ALF8 TCRs. Surface plasmon resonance binding curves of the ALF3 and ALF8 TCRs in solid phase tested against the GILGFVFTL-HLA-A*0201-M1 complex in fluid phase across a range of concentrations (0.78–200 μM). The mean ± SEM of two replicate assays is shown.

Figure S2

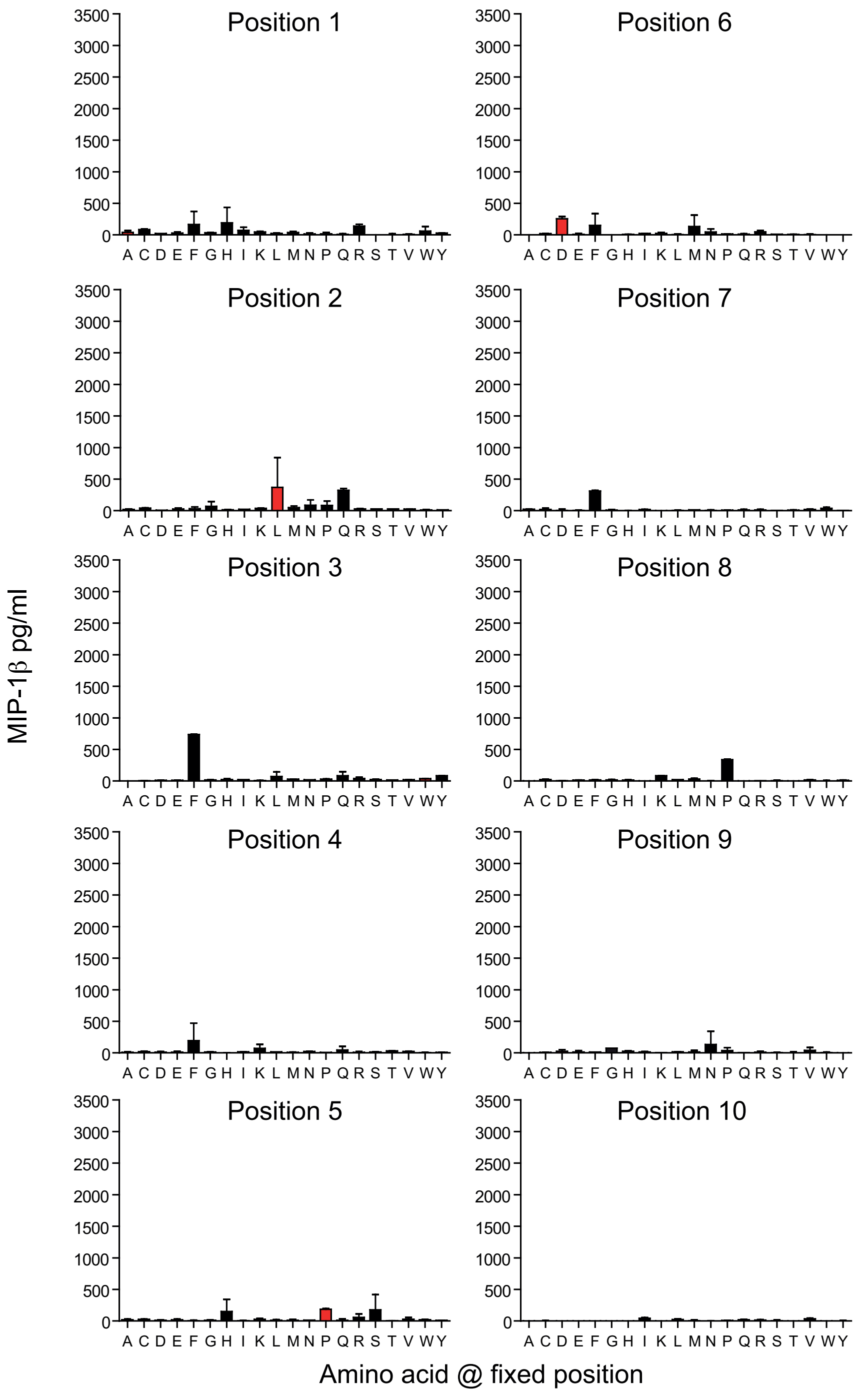

Figure S2: Combinatorial peptide library screen of the 1E6 CD8$^+$ T-cell clone in the absence of an intact pMHCI/CD8 interaction. 6 × 10^4 C1R-A*0201 D227K/T228A target cells were pulsed in duplicate with each mixture from a 10mer combinatorial peptide library (100 μM) for 2 hours at 37°C. 3 × 10^4 clonal 1E6 CD8$^+$ T-cells were then added and the plates were incubated overnight at 37°C. Supernatants were assayed for MIP-1β by ELISA. The index peptide sequence is shown as red bars in each position.

$CD8^+$ T-cell clone ID	No treatment (pEC_{50} MIP-1β)	0.25 µg/ml DK25 (pEC_{50} MIP-1β)	0.5 µg/ml DK25 (pEC_{50} MIP-1β)	1 µg/ml DK25 (pEC_{50} MIP-1β)
1E6	6.74921	-	-	-
3F2	6.02653	-	-	-
4C6	6.34537	-	-	-
ALF3	8.27954	7.9456	7.64974	7.76992
SB27	8.82749	8.81323	8.55271	8.72455
MCNLV	9.40681	8.82467	8.7935	8.60756

Table S1A: Functional sensitivity, measured by MIP-1β production, expressed as pEC_{50} (Figure 5)[1].

$CD8^+$ T-cell clone ID	No treatment (pEC_{50} lysis)	0.25 µg/ml DK25 (pEC_{50} lysis)	0.5 µg/ml DK25 (pEC_{50} lysis)	1 µg/ml DK25 (pEC_{50} lysis)
1E6	7.34425	-	-	-
3F2	7.06595	-	-	-
4C6	7.58755	-	-	-
ALF3	9.41053	8.69299	8.55927	8.62777
SB27	9.22728	9.06544	8.90326	8.44131
MCNLV	9.29825	9.11505	8.51186	8.24729

Table S1B: Functional sensitivity, measured by killing activity, expressed as pEC_{50} (Figure 6)[1].

$CD8^+$ T-cell clone ID	0.25 µg/ml DK25 (delta pEC_{50} MIP-1β)	0.5 µg/ml DK25 (delta pEC_{50} MIP-1β)	1 µg/ml DK25 (delta pEC_{50} MIP-1β)
1E6	>1.75	>1.75	>1.75
3F2	>1.03	>1.03	>1.03
4C6	>1.35	>1.35	>1.35
ALF3	0.33394	0.6298	0.50962
SB27	0.01426	0.27478	0.10299
MCNLV	0.58214	0.61331	0.79925

Table S1C: DK25-induced shifts in functional sensitivity, measured by MIP-1β production, expressed as delta pEC_{50} (Figure 5).

$CD8^+$ T-cell clone ID	0.25 µg/ml DK25 (delta pEC_{50} lysis)	0.5 µg/ml DK25 (delta pEC_{50} lysis)	1 µg/ml DK25 (delta pEC_{50} lysis)
1E6	>2.34	>2.34	>2.34
3F2	>2.07	>2.07	>2.07
4C6	>2.59	>2.59	>2.59
ALF3	0.71754	0.85126	0.78276
SB27	0.16184	0.32402	0.78597
MCNLV	0.78639	0.78639	1.05096

Table S1D: DK25-induced shifts in functional sensitivity, measured by killing activity, expressed as delta pEC_{50} (Figure 6).

1. Sprent P, Smeeton NC. Applied nonparametric statistical methods. *Chapman & Hall/CRC, London* (2007).
